# *Porphyromonas gingivalis* lipopolysaccharides act exclusively through TLR4 with a resilience between mouse and human

**DOI:** 10.1038/s41598-017-16190-y

**Published:** 2017-11-17

**Authors:** Brice Nativel, David Couret, Pierre Giraud, Olivier Meilhac, Christian Lefebvre d’Hellencourt, Wildriss Viranaïcken, Christine Robert Da Silva

**Affiliations:** 1 Université de La Réunion, INSERM, UMR 1188 Diabète athérothombose Thérapies Réunion Océan Indien (DéTROI), Saint-Denis de La Réunion, France; 2 CHU de La Réunion, Unité de soins intensifs neurologiques, Saint Pierre de La Réunion, France; 3 Université de La Réunion, CNRS UMR9192, INSERM U1187, IRD UMR249, Unité Mixte Processus Infectieux en Milieu Insulaire Tropical (PIMIT), Plateforme Technologique CYROI, Sainte-Clotilde, La Réunion, France

## Abstract

*Porphyromonas gingivalis* is a key bacterium in chronic periodontitis, which is associated with several chronic inflammatory diseases. Lipopolysaccharides from *P. gingivalis* (*Pg* LPS) can activate multiple cell types via the production of pro-inflammatory cytokines. The receptors for *Pg* LPS have initially been reported as TLR2, contrasting with the well-studied TLR4 receptor for *E. coli* LPS; this observation remains controversial since synthetic *Pg* lipid A activates TLR4 but not TLR2. Despite this observation, the dogma of *Pg* LPS-mediated TLR2 activation remains the basis of many hypotheses and result interpretations. In the present work, we aimed at determining whether TLR4 or TLR2, or both, mediate *Pg* LPS pro-inflammatory activity using *Pg* LPS with different grades of purity, instead of synthetic lipid A from *Pg* LPS. Here we show that *Pg* LPS 1) acts exclusively through TLR4, and 2) are differently recognized by mouse and human TLR4 both *in vitro* and *in vivo*. Taken together, our results suggest that *Pg* LPS activity is mediated exclusively through TLR4 and only weakly induces proinflammatory cytokine secretion in mouse models. Caution should be taken when extrapolating data from mouse systems exposed to *Pg* or *Pg* LPS to humans.

## Introduction

The innate immune system is responsible for an inflammatory response necessary for the recruitment and activation of the adaptive immune system. Toll Like Receptor (TLR), described for the first time in 1996^1^, plays an important role in the innate immune response, especially in the recognition of pathogen associated molecular pattern (PAMP) or microbial associated molecular pattern (MAMP). PAMP or MAMP binding to TLRs induces an activation of immune cells and activates several innate pathways, such as inflammation, phagocytosis, and cell death^2^.

TLR4 is the pattern recognition molecule (PRR) involved in the recognition of lipopolysaccharides (LPS)^3^, the major glycolipids present at the surface of gram-negative bacteria. Lipid A, found in the structure of LPS, is responsible for the toxicity of these bacteria^4^. In the blood flow, LPS is associated with LPS binding protein (LPB). This complex binds to CD14 and is then transferred to the TLR4/MD2 complex^5^. This association leads to the oligomerization of TLR4^6^. LPS from *Escherichia coli* (Ec LPS), a gram-negative bacteria, targets TLR4 and activates the NF-κB signaling pathway, leading to the secretion of inflammatory cytokines, such as Tumor Necrosis Factor α (TNF-α) and Interleukin 6 (IL-6), and chemokines, such as Monocyte Chemoattractant Protein 1 (MCP-1)^7^.

Another gram-negative bacterium, *Porphyromonas gingivalis* is a well-known bacteria responsible for the development of chronic periodontitis^8^. Periodontal disease is characterized by the infiltration of inflammatory cells in the gingiva, including macrophages. Recent studies have demonstrated a link between periodontitis and atherosclerosis^9,10^. Atherosclerosis is a multifactorial disease. Infection by *P. gingivalis* (whole bacteria or derived-products such as LPS or heat-shock proteins) has been identified as an important factor leading to an inflammatory response from endothelial cells and other cells of the vascular wall^11,12^. LPS from *P. gingivalis* (*Pg* LPS) is able to stimulate proinflammatory cytokine secretion leading to the recruitment of monocytes/macrophages into atherosclerotic plaques^13–17^. In C3H/HeJ mice, which are deficient for TLR4, *Pg* LPS exhibits an activity suggesting that its effect is mediated by TLR2^18,19^. However, both structural and functional studies of synthetic lipid A of *Pg* LPS have revealed that they are able to activate cells through TLR4 and not TLR2^20^; the TLR2 activity of *Pg* LPS might be attributed to a contaminant lipoprotein^21^. Consequently, the interaction of *Pg* LPS with TLR2 or TLR4 remains controversial^22^. This controversy is based on the purification method of LPS from bacteria. The original method of LPS extraction from bacteria is based on phenol-water extraction^23^. These phenol-water extracts mostly consist of LPS, but also contains other bacterial components, such as lipoprotein^24^. Despite these observations, the dogma of *Pg* LPS-mediated TLR2 activation remains the basis of many hypotheses and result interpretations^25–32^. In this work, we aimed at determining whether TLR4 and/or TLR2 are responsible for *Pg* LPS pro-inflammatory activity.

Discrepancies have been observed between species regarding their tolerance to LPS. For example, the dose of *Ec* LPS required to induce a septic shock in human is 1000–10,000-times lower than the dose used to induce cytokine production in mice^33,34^. In the present study, we also examined the pro-inflammatory properties of *Pg* LPS in mouse versus human using reporter cell lines.

*Pg* LPS appeared to mediate pro-inflammatory signaling exclusively through TLR4, whereas TLR2-dependent pathway was related to the presence of contaminants in LPS preparation. Finally, we observed that *Pg* LPS only induced a weak activation of the mouse relative to human TLR4.

## Material and Methods

### Drug, reagents and cell lines

Ultrapure *P. gingivalis* LPS (UP *Pg* LPS) (Invivogen, cat: tlrl-ppglps, lot: PPG-3701 and 3801), Standard *P. gingivalis* LPS (STD *Pg* LPS) (cat: tlrl-pglps, lot: LPG 37-01 and 38-01) were from InvivoGen (France). The STD *Pg* LPS were obtained by classical methods using hot-phenol extraction using the bacterial strain ATCC 33227 and UP *Pg* LPS preparations by enzymatic removal of lipoprotein from STD *Pg* LPS (Invivogen). Ultrapure lipopolysaccharide from *E. coli* O111:B4 (UP *Ec* LPS) (cat: tlrl-3pelps, lot: L3P 37-02) were from InvivoGen (France).

RAW264.7-Blue (RAW-Blue), HEK-Blue hTLR2-hCD14 (HEK-Blue hTLR2), HEK-Blue Null, HEK-Blue hTLR4-hCD14-hMD2 (HEK-Blue hTLR4), and HEK-Blue mTLR4-mCD14-mMD2 (HEK-Blue mTLR4) were from InvivoGen (France). BV2 was obtained as described previously^35^. J774.1 (TIB-67), 3T3-L1 (CL-173), bEnd.3 (CRL-2299), and RAW264.7 (TIB-71) were from ATCC (USA), and all of these cells line express functional TLR2 and TLR4. TNF-α was from R&D systems (USA). Except when indicated, all other reagents were from Sigma-Aldrich (France). NFkB activity was performed using Quanti-blue assay following the manufacturer’s protocols (InvivoGen).

### ESI-MS analysis

Lipid A from 10 µg of STD *Pg* LPS or UP *Pg* LPS were isolated by mild acid hydrolysis as previously^36^. The lyophilized Lipid A was suspended in 1000 µL of 1:100 CHCl3–CH3OH. ESI-MS, performed with a Q exactive plus instrument (Thermofischer), was used to evaluate negative ions of *P. gingivalis* lipid A for the 1417 m/z specie. Syringe pump was used to infuse the lipid A sample. Typical conditions were: infusion rate, 5 µL/min, spray voltage, 3.8 kV; capillary temperature, 320 °C. The 1417 m/z specie first isotope was normalized at 100% of abundance and intensity (NL) of this species was determined for each type of lipid A. This NL value represents the abundance 1417 m/z specie.

### Cell culture and treatment

All cell lines were cultured at 37 °C with 5% CO_2_ in Dulbecco’s Modified Eagle Medium (DMEM), supplemented with 10% (v/v) heat-inactivated fetal bovine serum (FBS) and 2 mmol.L^−1^ L-Glutamine, 1 mmol.L^−1^ sodium pyruvate, 100 U.mL^−1^ penicillin, 0.1 mg.mL^−1^ streptomycin, and 0.5 mg.mL^−1^ amphotericin B (PAN Biotech, Germany). Cells were plated at a density of 5.10^5^ cell.mL^−1^ in a 96-well plate. For treatment, the medium was replaced by a complete medium with different LPSs at 0 to 10 000 ng.mL^−1^ and incubated for 20 h. The culture media were collected and used for cytokine quantification by ELISA.

### Mouse stimulation and Human whole blood assy

All procedures were performed in accordance with guidelines for care and use of laboratory animals, approved by the ethics committee of CYROI (A974001). C57BL/6 mice purchased from Charles River Laboratory (France) were fed with a standard diet ad libitum^37^. Animals were randomly assigned to 1 of 4 groups (n = 3 per group, 2 males, and 1 female, 10-week old, approximately 25 g). Mice were anesthetized with Isoflurane®, and their body temperature was maintained at 37 °C with a heating pad. Analgesia was controlled by subcutaneous injection of Buprenorphine®. The throat was shaved, disinfected, and a 1–1.5 cm medio-lateral cervicotomy incision was made. The adjacent connective tissue was carefully stretched apart, exposing the external jugular vein. A tail vein catheter with a microinjection 30-gauge needle was used for intravenous injection. LPS solution was diluted with PBS at 100 µg.kg^−1^, and the injection volume was adjusted to 0.1 mL. After removing the needle and verifying the absence of bleeding, the skin was then sutured. The whole procedure took less than 5 min. After recovery from anesthesia, the animals were observed and moved to individual cages with water, food, and refinement for 3.5 h. After this observational period, mice were anesthetized with Isoflurane® and euthanized by intracardiac puncture. Blood was drawn into heparin tubes, centrifuged at 4000 g for 15 min and stored at −20 °C.

Human blood samples were obtained from 4 healthy volunteers without medication, after informed consent. Blood was sampled in lithium heparin-containing tubes, diluted 1:10 with 100 µL RPMI medium without FBS in a 96-well plate with different concentrations of LPS (from 0 to 10 000 ng.mL^−1^) for 20 h. The plate was then centrifuged at 1200 g for 5 min, and the supernatant was collected for the cytokine quantification by ELISA.

### ELISA/Cytokine assay

TNF-α, MCP-1, and IL-6 concentrations in cell culture supernatant, mouse plasma or human whole blood assay were assessed using ELISA kits (eBiosciences, Austria), following the manufacturer’s protocols. The following kit were used: Human TNF-α (cat: 88–7346) sensitivity 4 pg.mL^−1^, Human IL-6 (cat: 88–7066) sensitivity 2 pg.mL^−1^, Human MCP-1 (cat: 88–7399) sensitivity 7 pg.mL^−1^, Mouse TNF-α (cat: 88–7324) sensitivity 8 pg.mL^−1^, Mouse IL-6 (cat: 88–7064) sensitivity 4 pg.mL^−1^, Mouse MCP-1 (cat: 88–7391) sensitivity 15 pg.mL^−1^. The optical absorbance at 450 nm and the background at 570 nm were measured with a microplate reader (TECAN Sunrise, Austria). For cytokine analysis of *in vivo* and *ex vivo* experiments analysis, samples were tested with a serial dilution in triplicate for each point. The final concentrations were determined using the dilution within the standard curve.

### Statistical analysis

All values are expressed as mean +/−SEM resulting from at least three biologically independent experiments. Comparisons between different treatments have been analyzed by one-way ANOVA test with Tukey post-test or two-way ANOVA test followed by a Dunnett’s test. Values of p < 0.05 were considered statistically significant. All statistical tests were done using the software Graph-Pad Prism version 6.01 (San Diego, CA, USA). Degrees of significance are indicated in the figure captions as follow: *p < 0.05; **p < 0.01; ***p < 0.001; ns = not significant.

## Results

### *Pg* LPS analysis

TLR4 seems to be involved in the response to most LPS^38^. However, both TLR2 and TLR4 have been reported to be involved in the recognition of *Pg* LPS^30,39^. Since purification methods to obtain LPS are based on the lipid nature of this PAMP, lipoproteins, which are known to bind TLR2, may co-purify with LPS. Ultrapure LPS preparations, which consist of lipoprotein removal by enzymatic digestion after classical purification step, are commercially available.

**Figure 1:**
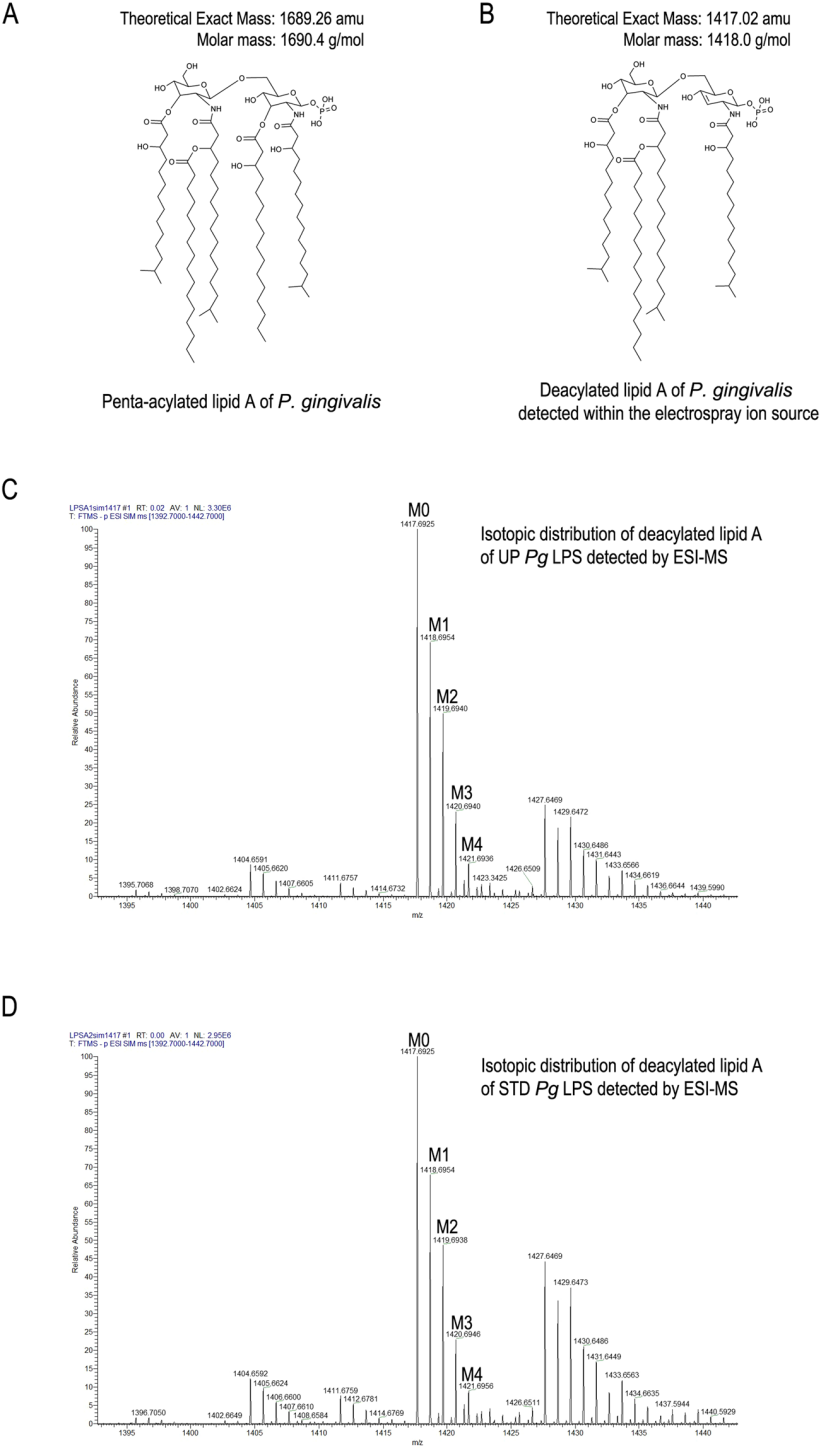
ESI-MS analysis of lipid A from *Pg* LPS. (**A**) Structure and molecular mass of penta-acylated lipid A (1690) of *P. gingivalis*. (**B**) Structure and molecular mass of penta-acylated lipid A was modified during electrospray ionization to generated a deacylated lipid A. Negative ions analysis of deacylated lipid A from STD *Pg* LPS (**C**) or UP *Pg* LPS (**D**) by ESI-MS.

We tested the hypothesis that lipoprotein contamination in standard *Pg* LPS (STD *Pg* LPS) preparations may be responsible for TLR2 activation by using both standard and ultrapure *Pg* LPS (UP *pg* LPS). *P.gingivalis* LPS with penta-acylated lipid A (PgLPS_1690_) are the main form involved in inflammation compared to the hexa-acylated lipid A for *E. coli* LPS^40–42^. We first characterized the lipid A recover from both *Pg* LPS by ESI-MS for their content in penta-acylated lipid A to rule-out the removal of PgLPS_1690_ during the repurification protocol designed to remove lipoproteins. With negative ions analysis by ESI-MS, the penta-acylated lipid A (m/z = 1690) were deacylated in the electrospray ion source^43^ and have a molecular mass of 1417 (Fig. 1A). We followed this ion for the two lipid A preparation, the same isotopic distribution (M0 to M7) for STD *Pg* LPS and UP *pg* LPS was found (Fig. 1B). The content of the two LPS preparation are comparable for PgLPS_1690_ as the intensity value for M0 are respectively NL = 2.95 × 10^6^ and NL = 3.30 × 10^6^ for STD *Pg* LPS and UP *pg* LPS_._ The endotoxic capacity of the three types of LPS using LAL assay was evaluated (Figure S1). Surprisingly, the endotoxic capacity of UP *Pg* LPS was 250-fold higher than that of STD *Pg* LPS by this method.

### Ultrapure *Pg* LPS induces cell activation via TLR4 but not TLR2

**Figure 2:**
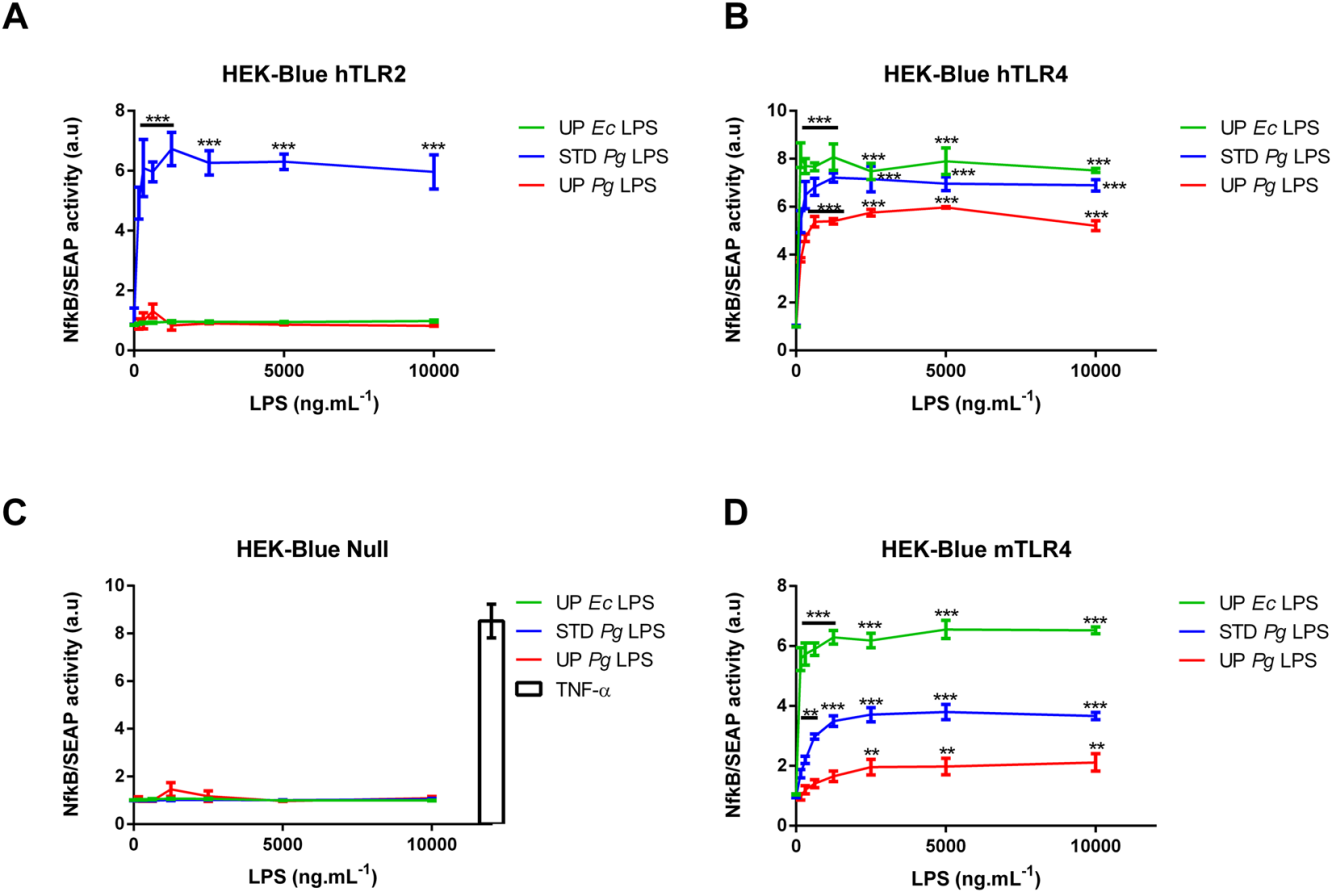
NFκB-AP1/SEAP activity in HEK-Blue cells stimulated with LPS. HEK-Blue Null, HEK-Blue hTLR2, HEK-Blue hTLR4 and HEK-Blue mTLR4 cells were treated with different doses of UP *Ec* LPS, STD *Pg* LPS or UP *Pg* LPS (0 to 10,000 ng.mL^−1^) for 20 h. TNF-α at 10 ng.mL^−1^ was used as positive control for HEK-Blue Null. SEAP activity in the culture supernatants was measured using Quanti-blue. Comparisons between different treatments have been analyzed by two-way ANOVA test followed by a Dunnett’s test. Data are expressed as mean ± SEM (n = 3 per group). *p < 0.05, **p < 0.01, ***p < 0.001 compared to 0 ng.mL^−1^ LPS.

HEK293 cells overexpressing human TLR2/CD14 (HEK-Blue hTLR2) or TLR4/MD2/CD14 (HEK-Blue hTLR4), which stably contain the NF-κB/AP1-SEAP reporter gene, were stimulated with different doses of standard *Pg* LPS, ultra-pure *Pg* LPS, and ultra-pure *E. coli* LPS (UP *Ec* LPS). These cell lines were incubated with increasing concentrations of LPS; activation was monitored via NF-κB/SEAP activity using Quanti-blue assay. Only STD *Pg* LPS significantly activated HEK-Blue hTLR2 cells (Fig. 2A). All three types of LPS were able to induce the hTLR4-signaling pathway but UP *Pg* LPS induced less activation than the others (p < 0.0001) (Fig. 2B). Of note, when both TLR2 and TLR4 were lacking (HEK-Blue Null), no activation was observed, whatever the type of LPS used (Fig. 2C).

### Mouse TLR4-MD2-CD14 complex poorly recognizes *Pg* LPS

Mice or mouse cell lines are widely used to evaluate the biological effects of *Pg* or *Pg* LPS. Previous studies suggest that mice are more tolerant to certain bacterial infection and exposure to LPS relative to human (i.e., *E. coli*). In contrast, mice are more competent to recognize *Leptospira interrogans* LPS^33,34^. Nothing has been reported about a potential resilience for Pg LPS in mice vs. humans. Therefore, we tested the capacity of mouse TLR4 to mediate *Pg* LPS response. HEK-Blue mouse TLR4 cells, which express TLR4/MD2/CD14 complex of mouse origin, were treated as described above. In contrast to HEK expressing human TLRs, a differential response dependent on the origin and the purity of LPS was observed (Fig. 2D). The maximal response was obtained with UP *Ec* LPS, whereas 50% and 70% loss of activity were observed with STD *Pg* LPS and UP *Pg* LPS, respectively (Fig. 2B and D). Mouse TLR4 seems to be less capable of recognizing *Pg* LPS relative to its human counterpart.

### Ultra-pure *Pg* LPS barely induce cytokine secretion by murine macrophages

**Figure 3:**
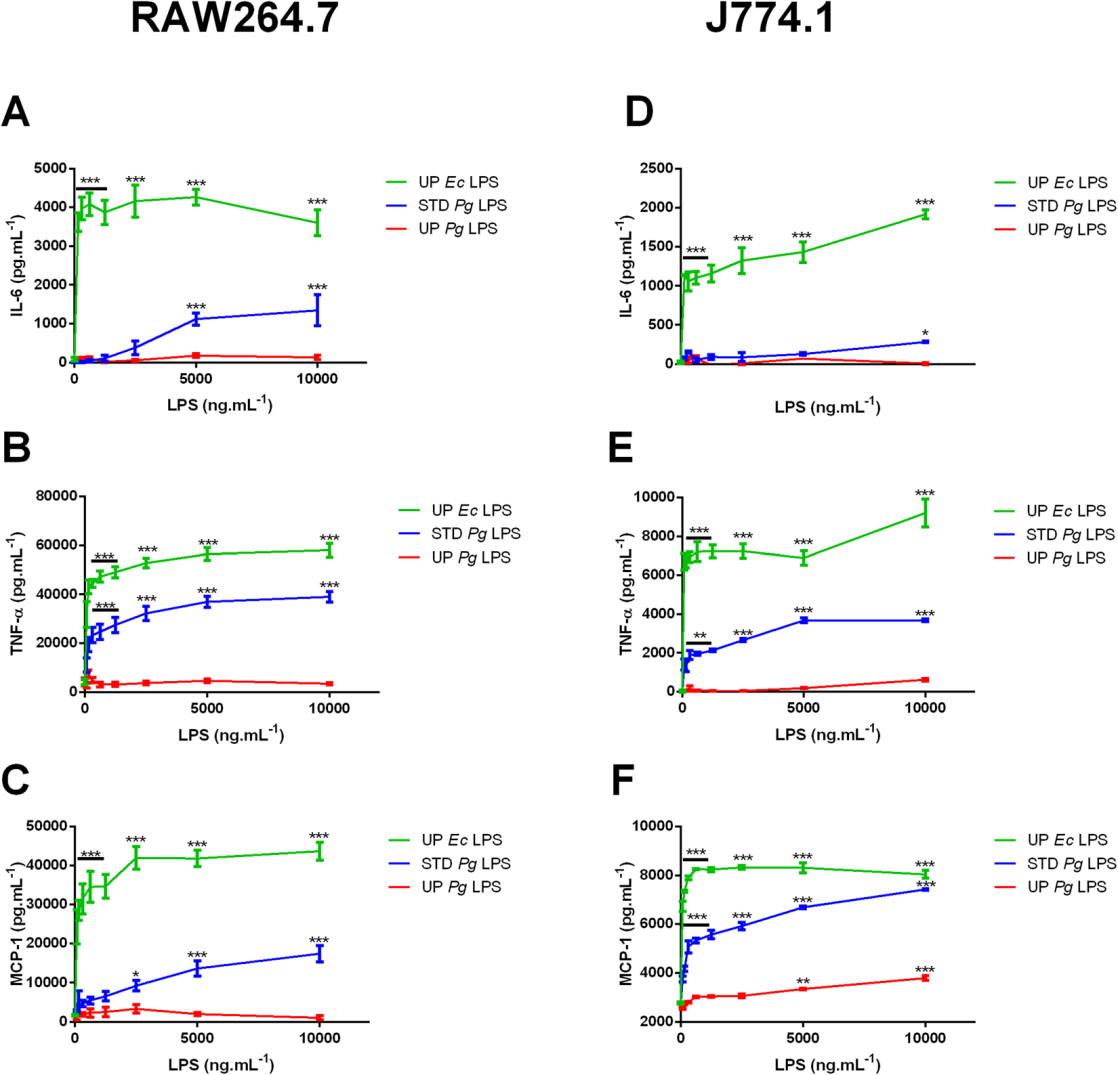
Cytokine release in RAW264.7 and J774.1 cells stimulated with LPS. RAW264.7 (Fig. 3A–C) and J774.1 (Fig. 3D–F) cells were treated with different doses of UP *Ec* LPS, STD *Pg* LPS or UP *Pg* LPS (0 to 10,000 ng.mL^−1^) for 20 h. IL-6 (Fig. 3A and D), TNF-α (Fig. 3B and E), and MCP-1 (Fig. 3C and F) levels were determined by ELISA in cell culture supernatants. Comparisons between different treatments have been analyzed by two-way ANOVA test followed by a Dunnett’s test. Data are expressed as mean ± SEM (n = 3 per group). *p < 0.05, **p < 0.01, ***p < 0.001 compared to 0 ng.mL^−1^ LPS.

To validate the weak response of mouse to *Pg* LPS, we quantified the production of pro-inflammatory cytokines induced by *Pg* LPS by two mouse macrophage cell lines: RAW264.7 (Fig. 3A–C) and J774.1 (Fig. 3D–F).

STD *Pg* LPS induced a dose-dependent secretion of IL-6 (Fig. 3A and D), TNF-α (Fig. 3B and E), and MCP-1 (Fig. 3C and F) in these two cell lines but to a lesser extent than UP *Ec* LPS. Interestingly, UP *Pg* LPS was not able to induce a strong production of pro-inflammatory cytokines, even at the highest dose tested compared to STD *Pg* LPS.

### Ultra-pure *Pg* LPS induces a weak inflammatory response in other murine cell lines: microglial cells, preadipocytes, and endothelial cells

**Figure 4:**
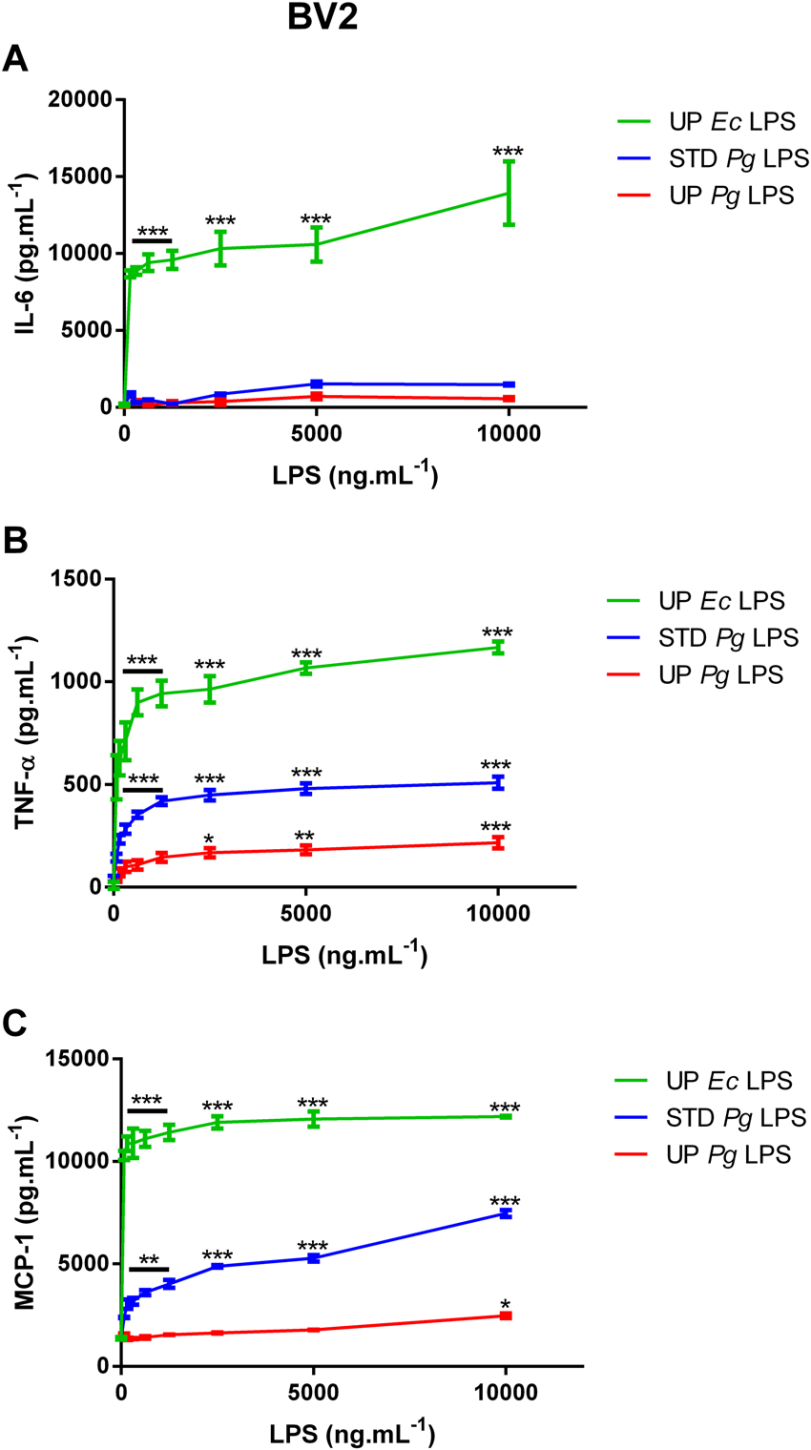
Cytokine release in BV2 cells stimulated with LPS. BV2 cells were treated with different doses of UP *Ec* LPS, STD *Pg* LPS or UP *Pg* LPS (0 to 10,000 ng.mL^−1^) for 20 h. IL-6, TNF-α, and MCP-1 levels were measured in the cell culture medium by ELISA. Comparisons between different treatments have been analyzed by two-way ANOVA test followed by a Dunnett’s test. Data are expressed as mean ± SEM (n = 3 per group). *p < 0.05, **p < 0.01, ***p < 0.001 compared to 0 ng.mL^−1^ LPS.

Since *Pg* LPS can be involved as a mediator of neuroinflammation^3,5^, similar experiments were performed using the mouse microglial cell line BV2 (Fig. 4). As observed with macrophage cell lines, UP *Ec* LPS induced a marked pro-inflammatory cytokine secretion. The concentration of secreted IL-6 (Fig. 4A) was not significantly different from the basal levels after stimulation with both STD and UP *Pg* LPS. In contrast, TNF-α (Fig. 4B) and MCP-1 (Fig. 4C) secretion was clearly stimulated by STD *Pg* LPS but only weakly by UP *Pg* LPS when used at high doses.

**Figure 5:**
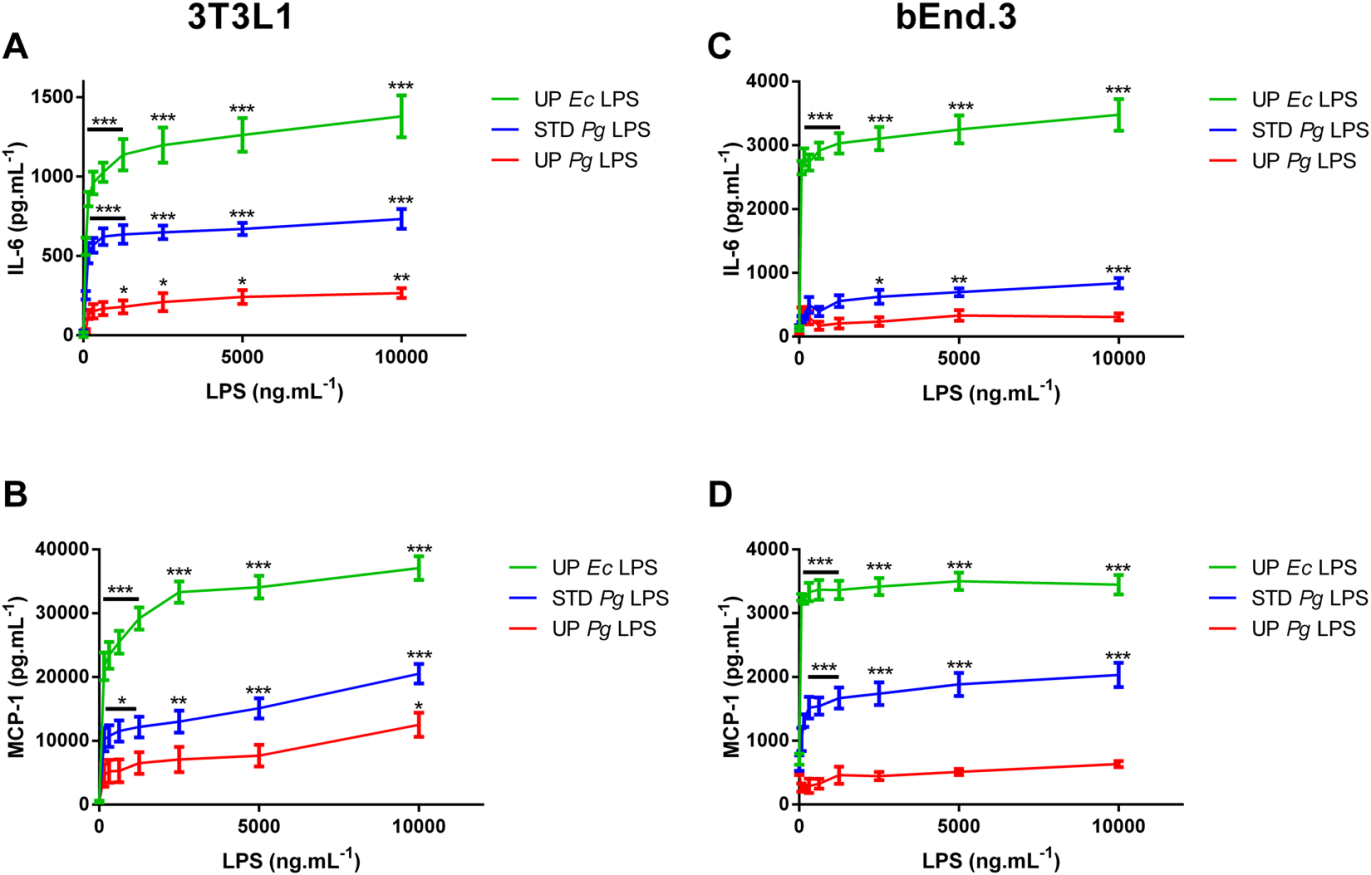
Cytokine release in 3T3-L1 and bEnd.3 cells stimulated with LPS. 3T3-L1 (Fig. 5A–C) and bEnd.3 (Fig. 5D–F) cells were treated with different doses of UP *Ec* LPS, STD *Pg* LPS or UP *Pg* LPS (0 to 10,000 ng.mL^−1^) for 20 h. TNF-α, IL-6, and MCP-1 concentrations were measured in the cell culture supernatants by ELISA. Comparisons between different treatments have been analyzed by two-way ANOVA test followed by a Dunnett’s test. Data are expressed as mean ± SEM (n = 3 per group). *p < 0.05, **p < 0.01, ***p < 0.001 compared to 0 ng.mL^−1^ LPS.

Even if immune phagocytic cells are specialized to sense PAMPs, other cell types are also sensitive to these factors. For example, pre-adipocytes and adipocytes express functional TLR2 and TLR4^44–46^. Mouse pre-adipocyte cell line 3T3L1 (Fig. 5A,B) was stimulated with increasing concentrations of the different LPS, and cytokine secretion was then measured by ELISA. UP *Pg* LPS induced a weak, but statistically significant, dose-dependent cytokine secretion of IL-6 (Fig. 5A) and MCP-1 (Fig. 5B). As shown above, UP *Pg* LPS was much less effective than STD *Pg* LPS in stimulating the secretion of pro-inflammatory cytokine in this cell line. No TNFα secretion were detectable upon induction with all 3 LPS as previously describe^44,45,47^.

Endothelial cells are also able to recognize LPS and elicit a pro-inflammatory pattern^48^. To test the capacity of mouse endothelial cells in sensing *Pg* LPS, we stimulated brain endothelial mouse cell line, Bend3, with different LPS and then measured cytokine secretion by ELISA. UP *Pg* LPS neither induced IL-6 nor MCP-1 secretion (Fig. 4C and D). STD *Pg* LPS was able to induce the secretion of pro-inflammatory cytokines (IL-6 and MCP-1) in this cell line but to a lesser extent than UP *Ec* LPS, used as a positive control. No TNFα were detectable with the 3 LPS as support by previous observation^49^. These results suggest that mouse non-immune cell lines, such as pre-adipocytes and endothelial cells, weakly recognize and respond to *Pg* LPS.

### *Pg* LPS induces a low pro-inflammatory response in mice

**Figure 6:**
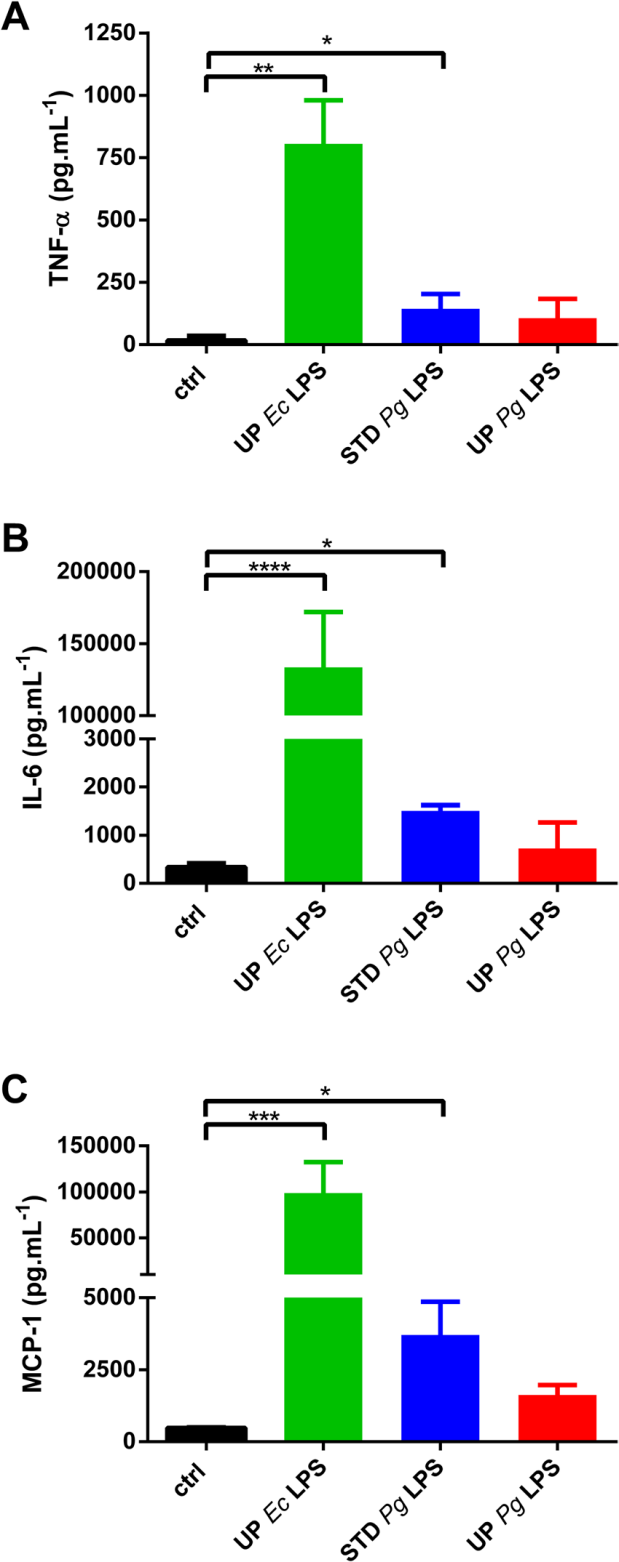
Cytokine release in mice stimulated with LPS. Mice were treated with 100 µL of UP *Ec* LPS, STD *Pg* LPS or UP *Pg* LPS (100 µg.kg^−1^) for 3.5 h. Control mice received saline. Plasmatic levels of TNF-α, IL-6, and MCP-1 were measured by ELISA. Comparisons between different treatments have been analyzed by one-way ANOVA test with Tukey post-test. Data are expressed as mean ± SEM (n = 3 per group). *p < 0.05, **p < 0.01, ***p < 0.001.

**Figure 7:**
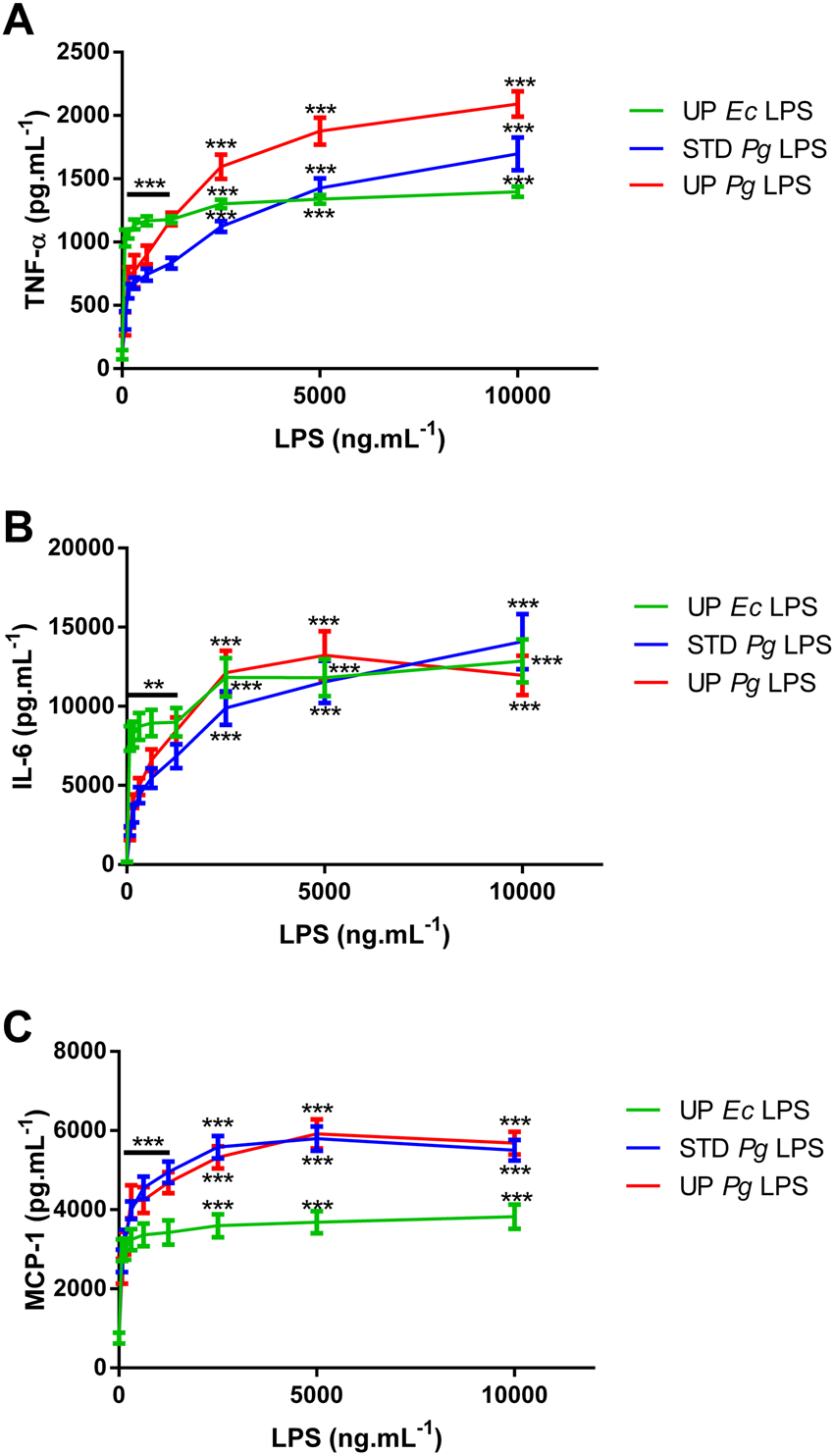
Cytokine production in human whole blood cells challenged with LPS. Human whole blood cells were treated with different doses of UP *Ec* LPS, STD *Pg* LPS or UP *Pg* LPS (0 to 10,000 ng.mL^−1^) for 20 h. TNF-α, IL-6, and MCP-1 levels were measured by ELISA in culture supernatants. Comparisons between different treatments have been analyzed by two-way ANOVA test followed by a Dunnett’s test. Data are expressed as mean ± SEM (n = 3 per group). *p < 0.05, **p < 0.01, ***p < 0.001 compared to 0 ng.mL^−1^ LPS.

To confirm that TLR4/MD2/CD14 could be less efficient at binding *Pg* LPS in mice than in humans, we tested the pro-inflammatory action of *Pg* LPS *in vivo* in mice. Mice were intravenously injected with a single dose of LPS at 100 µg.kg^−1^. Blood was collected after 3.5 h, and quantification of TNF-α, IL-6 and MCP-1 proteins was performed by ELISA (Fig. 6). Unlike for UP *Ec* LPS, only a weak response reflected by low cytokine production was obtained by STD *Pg* LPS injection. UP *Pg* LPS injection did not produce any significant difference in cytokine production versus saline-injected mice. To better characterize resilience between mouse and human for *Pg* LPS recognition, we performed a human whole blood assay with the three types of LPS. Since the above-mentioned results suggest that *Pg* LPS recognition and subsequent inflammatory response is specie-dependent (mouse vs human), we performed human whole blood assay to test *ex-vivo*, the human response to *Pg* LPS. Whole blood cells obtained from human donors have been treated with different concentrations of the three LPS for 20 h (Fig. 7). Plasma IL-6, TNF-α, MCP-1 concentrations was measured by ELISA after centrifugation. Stimulation with all 3 LPS resulted in an enhanced secretion of TNF-α, IL-6, and MCP-1 by human whole blood cells, in a dose-dependent manner. All of these observations confirm the resilience between human and mouse.

## Discussion

*Pg* is a gram negative bacterium associated with the pathogenesis of periodontitis^50^. Periodontitis has been linked to numerous low-grade inflammatory diseases, such as obesity^51,52^, atherosclerosis^11,12,15^, and neuroinflammatory diseases (Alzheimer’s)^53–55^. LPSs are the major inflammatory mediators for gram negative bacteria. *Pg* LPS is able to elicit cell inflammatory responses via interaction with TLRs^56^. The lipid A component is responsible for LPS binding to MD2, the resulting complex then binds to TLR4 and triggers a signaling cascade leading to the production and secretion of pro-inflammatory cytokines. Even if TLR4 is described as the canonic receptor for LPS, *Pg* LPS was reported to induce a pro-inflammatory response in C3H/HeJ mice which are deficient for TLR4. TLR2-CD14 was reported to be the major complex involved in the recognition of *Pg* LPS^18,19^. Since synthetic *Pg* lipid A trans-activated cells through TLR4^20^, *Pg* LPS TLR2 binding and subsequent cell activation were attributed to contaminant bacterial lipoproteins^21^. A recent study suggests that *Pg* LPS can interact with both TLR2 and TLR4^22^.

LPS extraction and purification methods are based on organic extraction using phenol^23^ that may lead to co-purification of lipoproteins. Ultra-pure *Pg* LPS, which is commercially available, is obtained after lipoprotein removal from the LPS preparation by an additional enzymatic digestion step. Using this ultra-pure *Pg* LPS, we tested whether TLR2 or TLR4, or both, were involved in *Pg* LPS recognition.

Whereas hTLR4 showed the same sensitivity towards the different LPS, neither UP *Pg* LPS nor UP *Ec* LPS were able to activate TLR2. Since it has been shown that the signaling via TLR2 could be due to PG1828, a lipoprotein synthesized by *Pg* ^57,58^, NF-κB activation observed in the presence of STD *Pg* LPS could be attributed to contamination by lipoproteins.

Nevertheless, UP *Pg* LPS was much more efficient in the human TLR4/MD2/CD14 system relative to that observed for the mouse complex. This result was confirmed by challenging mice or human whole blood with *Pg* LPS (Figs 6 and 7).

We also observed that STD *Pg* LPS is a stronger NF-κB activator than UP *Pg* LPS in both human and mouse TLR4 response. This differential recognition could be due to the structural heterogeneity between *Pg* LPS and *Ec* LPS, mainly of the lipid A structure^38^. *Pg* contains several different structures for the lipid A^22,42,59,60^. Different lipid A can also be obtained according to the extraction procedure^22^. It has been reported that lipid A species with an m/z 1,690 were the dominant structures found among multiple lipid A species from *Pg*, and this species are present in both STD *Pg* LPS and UP *Pg* LPS (Fig. 1B,C). This lipid A is a penta-acylated lipid A and is known to induce inflammation, whereas the other forms do not^40,41^.

Using LAL assay, endotoxin activity of UP *Pg* LPS was 250-fold higher than STD *Pg* LPS. Endotoxin activity measured by LAL assay can be different from the pro-inflammatory activity^38^. The extent of activation of the *Limulus* cascade cannot be directly correlated with the pyrogenic potential for humans and contaminant in STD *Pg* LPS can maybe interfere with the LAL assay. But as UP *Pg* LPS have higher endotoxin activity and Lipid A 1690 are present in both preparation, these observations suggest that lipoproteins present in STD *Pg* LPS may potentiate *Pg* LPS action through TLR4.

**Figure 8:**
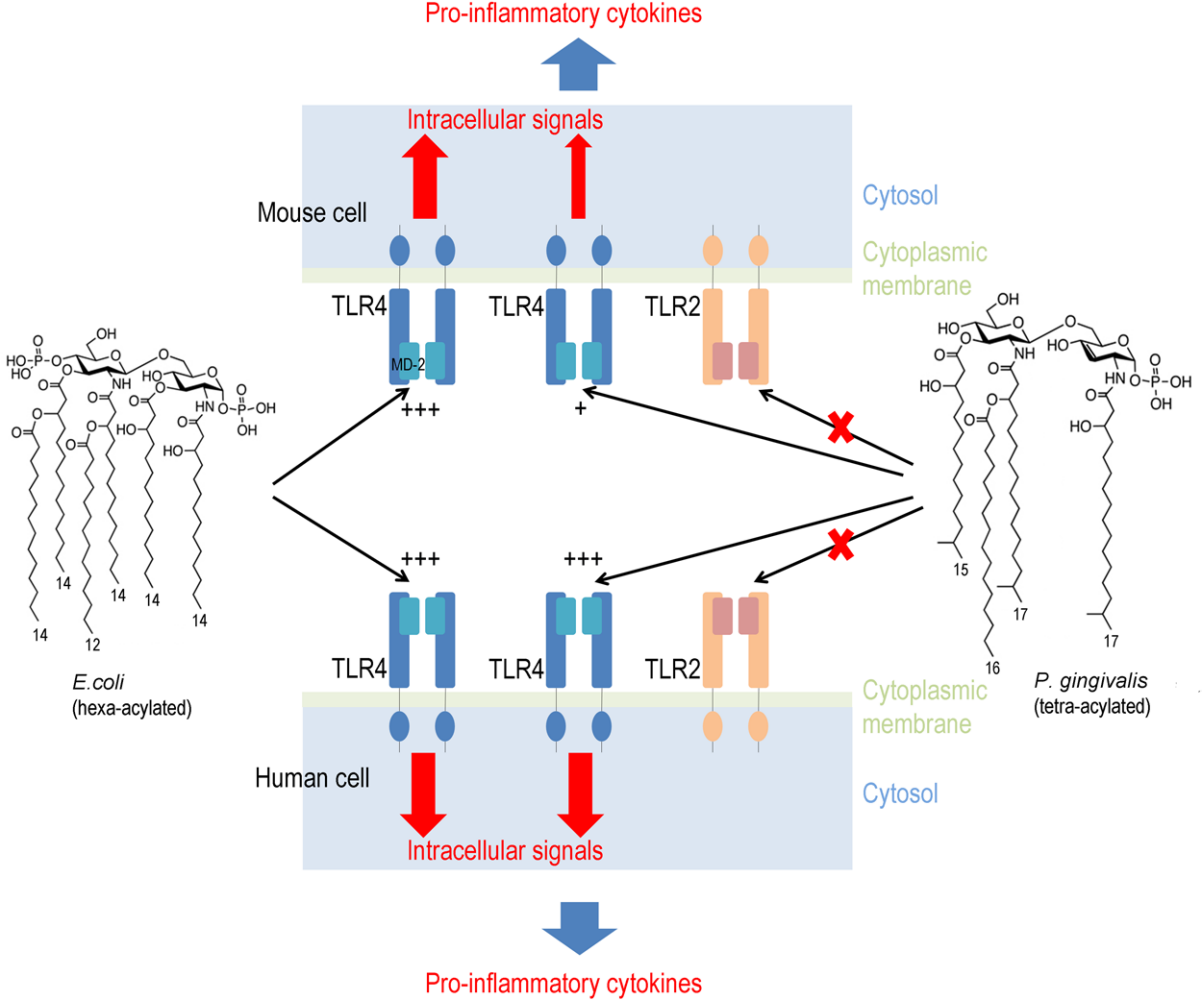
Differential recognition of lipid A on Pg LPS and *Ec* LPS. LPS recognition by TLR4 is based on the lipid A structure. Lipid A is different between *Ec* LPS and *Pg* LPS. Lipid A binding to MD2 induces dimerization of TLR4, activates the downstream signaling pathways and then leads to the secretion of pro-inflammatory cytokines. Our result suggests that recognition of lipid A structures by mouse and human TLR4 is different. Hexa-acylated *E. coli* lipid A is recognized as a strong agonist by both mouse and human TLR4, and induced an important production of pro-inflammatory cytokines. Penta-acylated *P. gingivalis* lipid A is recognized as an agonist by human TLR4 through MD2, and induces a production of proinflammatory cytokines, whereas it was weakly recognized by mouse MD2 and induces a weak production of pro-inflammatory cytokines. Penta-acylated *P. gingivalis* lipid A does not interact with TLR2.

Interestingly, modification of the Hexa-acylated lipid A of *E. coli* to penta-acylated lipid A showed reduced immunogenicity^61^. As UP *Ec* LPS induced the same NF-κB activity with mouse and human TLR4/MD2/CD14, we can hypothesize that mouse MD2 may have a better affinity for the Hexa-acylated lipid A relative to the penta-acylated form. Thus, mouse MD2 affinity for LPS may be more sensitive to acylation levels of lipid A as compared to human MD2 (Fig. 8). In humans, a recent study suggested that certain *TLR4* polymorphisms in *P. gingivalis* carriers favored alveolar bone loss^62^. Critical differences between human and mouse TLR4 in the amino acid sequence involved in recognition of MD2-LPS complex may also support our observation.

More importantly, mice or mouse cell models are widely used to investigate the role of *Pg* and *Pg* LPS in different diseases. We clearly demonstrated that *Pg* LPS barely activate mouse cells. The difference between mouse and human cell models to be activated by *Pg* LPS could be related to differences in expression levels and differential affinity of MD2 to penta-acylated *Pg* lipid A. Altogether, our results show that *Pg* LPS act through TLR4 and that mouse cells are less sensitive to *Pg* LPS than human cells. These results highlight that the use of mouse models for testing *Pg* and *Pg* LPS in different pathological situations might dangerously underestimate the role of these bacteria and associated endotoxins in human pathology.

## Electronic supplementary material

Supplementary Information
