# Supplementary material for: Porphyromonas gingivalis lipopolysaccharides act exclusively through TLR4 with a resilience between mouse and human
Source: Sci Rep. 2017 Nov 17;7:15789. doi: 10.1038/s41598-017-16190-y (PMC5693985; doi:10.1038/s41598-017-16190-y)
Supplement: Supplementary file 1 — Supplementary Information [file 41598_2017_16190_MOESM1_ESM.pdf]

Supplemental Figure 1. Ultra-pure Pg LPS has a significant endotoxin activity.

Porphyromonas gingivalis lipopolysaccharides act exclusively through TLR4 with a resilience between mouse and human.

Brice Nativel, David Couret, Pierre Giraud, Olivier Meilhac, Christian Lefebvre d'Hellencourt, Wildriss Viranaïcken and Christine Robert Da Silva.

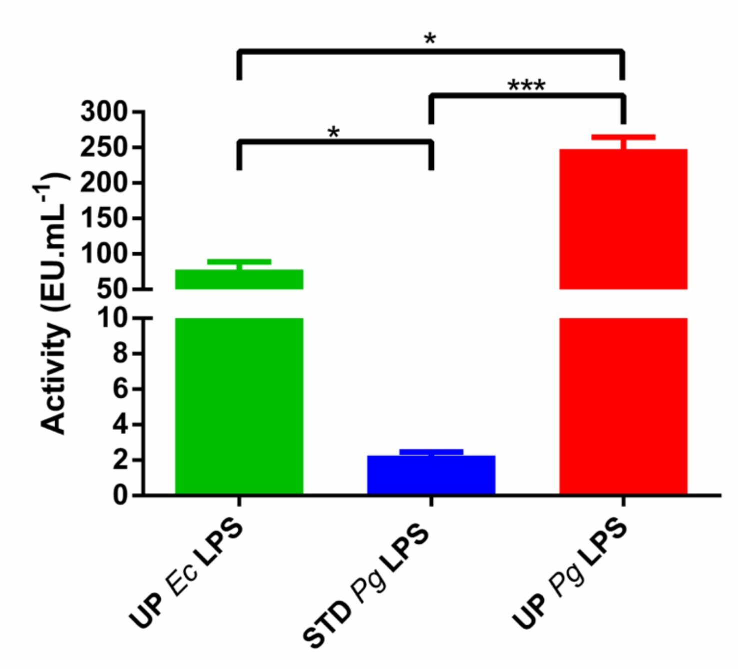

Endotoxin levels were determined in LPS using a Limulus Amebocyte Lysate (LAL) assay (Hycult biotech, The Netherlands). The assay was performed according to the manufacturer's protocol, and measurable levels of LPS were expressed as EU.ml^{-1}. UP *Ec* LPS were used as reference and normalized at 100%.

Comparisons between different treatments have been analyzed by one-way ANOVA test with Tukey post-test. Data are expressed as mean ± SEM (in triplicate). *p<0.05, **p<0.01, ***p<0.001
